# Exploring slow-phase eye velocity and nystagmus duration following caloric irrigation in Ménière’s disease

**DOI:** 10.1177/09574271251413135

**Published:** 2025-12-29

**Authors:** Jonathan Dion, Issam Saliba, Francois Champoux, Timothy E Hullar, Maxime Maheu

**Affiliations:** 1 School of Speech Language Pathology and Audiology, 5622 University of Montreal, Montreal, QC, Canada; 2 Institut universitaire sur la réadaptation en déficience physique de Montréal (IURDPM), Pavillon Laurier, CIUSSS du Centre-Sud-de-l'île-de-Montréal, Montreal, Canada; 3 Division of Otorhinolaryngology and Head & Neck Surgery, 5622 University of Montreal, Montreal, QC, Canada; 4 National Center for Rehabilitative Auditory Research (NCRAR), Veterans Affairs Portland Health Care System, Portland, OR, USA

**Keywords:** vestibular, Ménière’s disease, videonystagmography

## Abstract

**Introduction:**

In the early years following the development of the caloric test, one of the parameters used to quantify vestibular function was nystagmus duration. A common finding in patients with Meniere’s disease (MD) is the presence of unilateral caloric weakness, despite normal results on the video head impulse test (vHIT). It has been proposed that the apparent caloric loss could be due to the creation of local flow facilitated by canal distention. If the nystagmus duration is linked to the hydrostatic effect, one could hypothesize that MD patients would demonstrate shorter slow-phase velocity (SPV) duration.

**Objective:**

The aim of the study was to examine SPV and the duration of nystagmus following caloric testing in MD patients.

**Method:**

24 participants (12 controls; 12 MD) were assessed using bithermal, bilateral caloric stimulation. To control for possible side differences, two subgroups were analyzed: MD-ipsilateral (affected ear); MD-contralateral (unaffected ears). Ears in the control group were side matched with ears in the MD groups and the terminology control-ipsilateral or control-contralateral was used to represent this matching.

**Results:**

Significant differences were observed for SPV between controls-contralateral and MD-ipsilateral ears (cold: p ≤ 0.001; warm: p = 0.02) and between controls-ipsilateral and MD-ipsilateral ears (cold: p ≤ 0.001; warm: p = 0.04). Significant SPV differences were observed between MD-contralateral and MD-ipsilateral ears (cold: p ≤ 0.001; warm: p = 0.03). However, no differences were found between groups in the duration of nystagmus following cold or warm irrigation (cold: p = 0.2; warm: p = 0.33).

**Conclusion:**

Results support previous literature suggesting SPV can be affected in patients with MD even if vHIT gain is normal. However, mechanisms that influence the SPV seem to differ from those for the nystagmus duration. Indeed, nystagmus duration seems to remain unaffected in MD patients with vHIT-caloric dissociation, suggesting that it may be controlled by other factors.

## Introduction

A common finding in patients with Meniere’s disease (MD) is the presence of unilateral caloric weakness, despite normal results on the video head impulse test (vHIT) [for a review: Refs. 1,2]. Two hypotheses have been proposed to explain this dissociation. First, the isolated insensitivity to caloric stimulation might reflect a selective loss of low-frequency responsiveness of the semicircular canals. Tsuji et al.^3^ demonstrated that MD patients had a significant decrease in type II hair cells but a relatively normal number of type I hair cells. Type I hair cells have been shown to be innervated by irregular afferents, as opposed to type II hair cells, which are mostly innervated by regular afferents.^4^ Irregular afferents are more sensitive to high-acceleration (transient) stimulation, whereas regular afferents respond more specifically to sustained stimulation.^5,6^ Thus, the dissociation between vHIT and caloric results could also be associated with the influence of MD on type II versus type I hair cells.^7^ Second, McGarvie et al.^8^ proposed that the apparent caloric loss could be due to local flow facilitated by canal distention. This local flow would attenuate the hydrostatic effect caused by density differences and reduce caloric responses. Understanding the relative contribution of these two (not necessarily mutually exclusive) possibilities might be helpful in diagnosing and treating vestibular symptoms in patients with Meniere’s.

Caloric stimulation consists of positioning the patient’s head 30° relative to the horizontal, so the lateral semicircular canal is vertical, and introducing warm or cold air or water in the external ear canal.^9^ It is generally accepted that, in addition to local hydrostatic effects,^8^ at least part of the reaction is likely to be a result of direct thermal effect on the vestibular end organs or afferent neurons.^10^ Warm caloric irrigation is thought to induce excitation (increase neural firing) and cold caloric irrigation to induce inhibition (reduce neural firing) of the lateral canal of the stimulated ear only.^11^

In the early years following the development of the caloric test, the parameters that were used to quantify vestibular function following caloric stimulation were the duration of the nystagmus and the number of beats of nystagmus.^12^ Several studies investigated the relation between slow-phase eye velocity (SPV) and duration but failed to demonstrate any significant correlation.^13,14^ The absence of correlation between these two parameters led authors to propose that they are a manifestation of two separate mechanisms^15^ such that the duration of the nystagmus is related to the thermal properties of the temporal bone and the SPV is related to the physiological function of the semicircular canal during caloric stimulation.^13,14,16^

If Meniere’s affects the thermal properties of the labyrinth as suggested by McGarvie et al.,^8^ we might expect that both the duration of nystagmus and its SPV to be affected in Meniere’s patients. Therefore, the main objective of the present study was to compare the slow-phase eye velocity and the duration of the nystagmus in the identification of Meniere’s disease.

## Method

### Participants

Twenty-four participants (n = 12 controls; n = 12 unilateral Meniere’s) were assessed using bithermal, bilateral caloric stimulation. Meniere’s participants fulfilled probable (n = 7) or definite (n = 5) Meniere’s diagnosis criteria.^17^ Caloric-vHIT dissociation (e.g., vHIT: normal VOR gain of the lateral canals; caloric: unilateral caloric weakness) was a criteria for MD participants given that it has been identified to be present in hydropic ears (Kaci et al., 2020). Meniere’s participants were divided into affected (7 right ears and 5 left ears) and unaffected ears (5 right ears and 7 left ears). To control for possible side differences, controls were also subdivided in two groups paired (number of right vs left ears) with the ipsilateral and contralateral ears of Meniere’s group (controls-ipsilateral; controls-contralateral). None of the participants from the healthy group reported postural instability or vestibular problems. This study was approved by the ethic committee of the University of Montreal (CERC-20-072-P) and all participants gave written informed consent prior to testing.

### Protocol

First, to ensure normal function of the middle ear (i.e., avoid presence of otitis media or tympanic membrane perforation), otoscopy and tympanometry were performed on all participants prior to caloric testing. Participants with abnormal results were excluded.

Second, a vHIT (ICS Impulse, Otometrics, Taastrup, Denmark) was performed according to the usual protocol,^18^ to assess lateral semicircular canal function. A gain between 0.8 and 1.2 with absence of significant catch-up saccades was considered normal.^19^ None of the participants from the healthy groups had abnormal results and MD participants with abnormal vHIT were excluded.

Third, on the same day as the vHIT procedure, participants performed the vestibular caloric test using open loop warm and cold water irrigation (Aquastim, Interacoustics, Middelfart, Denmark) as previously described.^20^ The temperature of irrigation was 44°C for warm and 30°C for cold irrigations, each lasting 30 s with a total volume of 250 ml.^21^ Participants reclined at an angle of 30° relative to the horizontal, placing the lateral canals parallel to gravity.^22^ The nystagmus slow-phase velocity was measured using videonystagmography (VNG; VNS 3X, Synapsys, Marseille, France) goggles at a sampling rate of 33.33 Hz. The threshold for unilateral caloric weakness was 20%.

### Analysis

Individual tracings were visually inspected, ensuring that the appropriate SPV peak was selected. SPV was determined using proprietary software (Synapsys, Marseille, France) before being exported and analyzed in Matlab (R2020a) using a custom script. Peak SPV was determined by averaging the three consecutive highest values of SPV. Duration was defined as the time over which the SPV was greater than half its maximum value and greater than 2°/s, which was considered the noise floor (Figure 1).^14^ Duration was not calculated for three participants in MD-affected ears as the SPV peak failed to exceed 2°/s.

**Figure 1.**
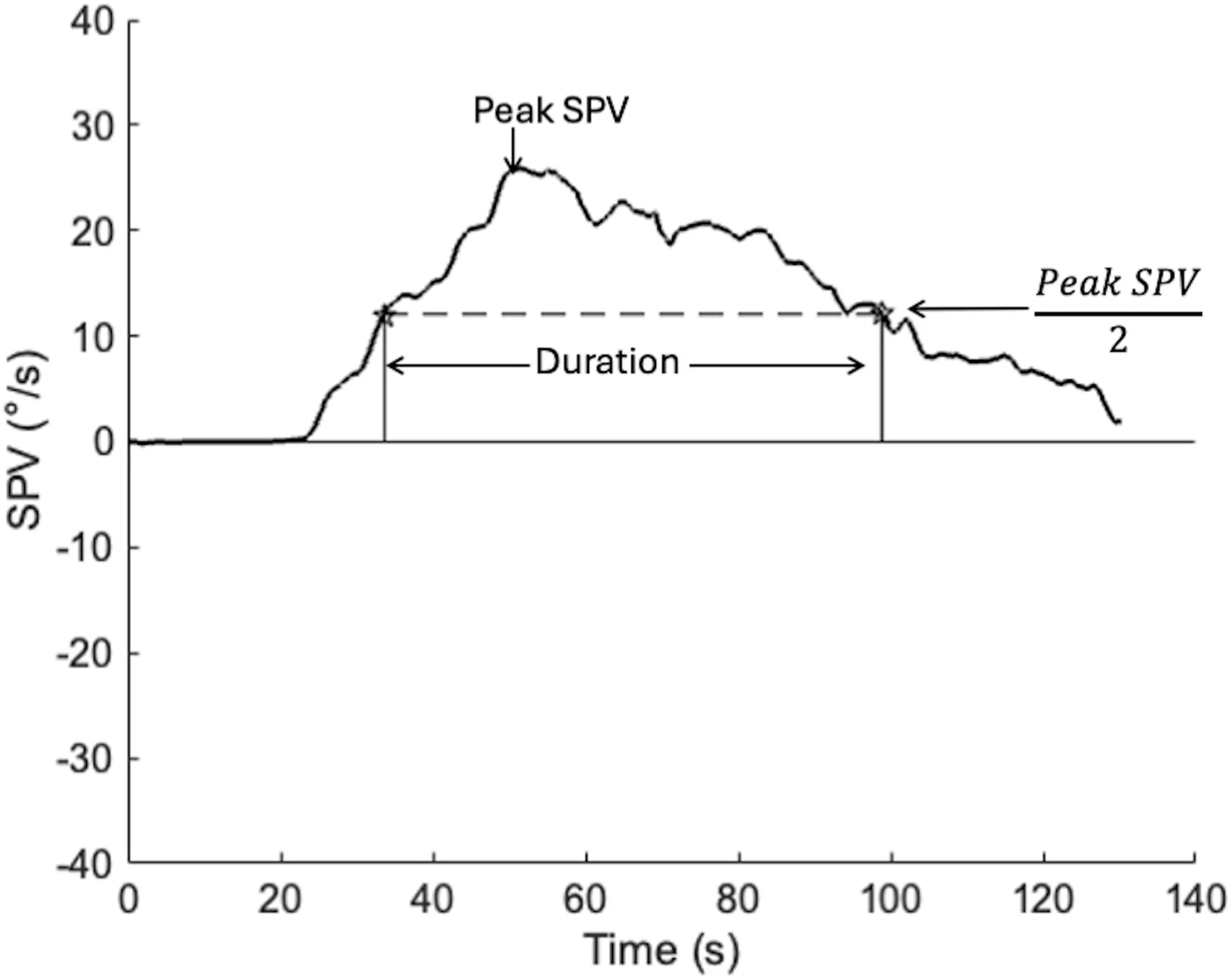
Representation of the calculation used for nystagmus duration and identification of peak SPV on a representative participant.

Repeated measures ANOVA with 2 patient groups (controls; MD) × 2 ears (ipsilateral; contralateral) was used to compare the maximum SPV and duration. A second repeated measure ANOVA with 2 patient groups (controls; MD) × 2 ears (ipsilateral; contralateral) was used to compare the VOR gain between groups and within ears. Post-hoc t-tests using a Bonferroni correction were used to assess differences between groups and ears. Statistical analysis was conducted in JASP (version 0.18.3).

## Results

No significant age difference was observed between the control and Meniere’s groups (Table 1). Mean and standard deviation for SPV and duration of each group is provided in Table 2. VOR gain analysis yielded neither a significant group difference (F (1, 22) = 1.28; p = 0.27) nor any significant ear difference (F (1, 22) = 2.39; p = 0.14). Mean and standard deviation of VOR gains from each group are provided in Table 2.

**Table 1.** Demographic informations for each group. No significant age difference was observed between both the control and Meniere’s group.

	n	Mean age (min–max)	Gender (M/F)	Number of ears (left/right)
Controls	12	50 [23–76] years old	6/6	Affected ears: 5/7
				Unaffected ears: 7/5
Meniere	12	52 [37–66] years old	5/7	Affected ears: 5/7
				Unaffected ears: 7/5

**Table 2.** Mean and standard deviation for slow phase eye velocity (SPV) and duration of each group.

	Warm SPV (± SD)	Warm duration (± SD)	Cold SPV (± SD)	Cold duration (± SD)	VOR gain (± SD)
Controls					
Ipsilateral	31.95°/s ± 15.36	62.90s ± 18.30	21.76°/s ± 7.61	70.19s ± 13.49	0.93 ± 0.07
Contralateral	34.10°/s ± 13.95	60.81s ± 15.00	20.70°/s ± 6.40	71.58s ± 21.00	0.94 ± 0.07
Meniere					
Ipsilateral	15.15°/s ± 12.48	60.07s ± 25.51	8.53°/s ± 5.86	61.27s ± 24.85	0.89 ± 0.06
Contralateral	24.38°/s ± 13.76	52.13s ± 15.96	17.15°/s ± 6.91	62.83s ± 12.62	0.94 ± 0.08

Caloric results of a representative participant from each group (Meniere’s and control) for both ears (ipsilateral and contralateral) are shown in Figure 2.

**Figure 2.**
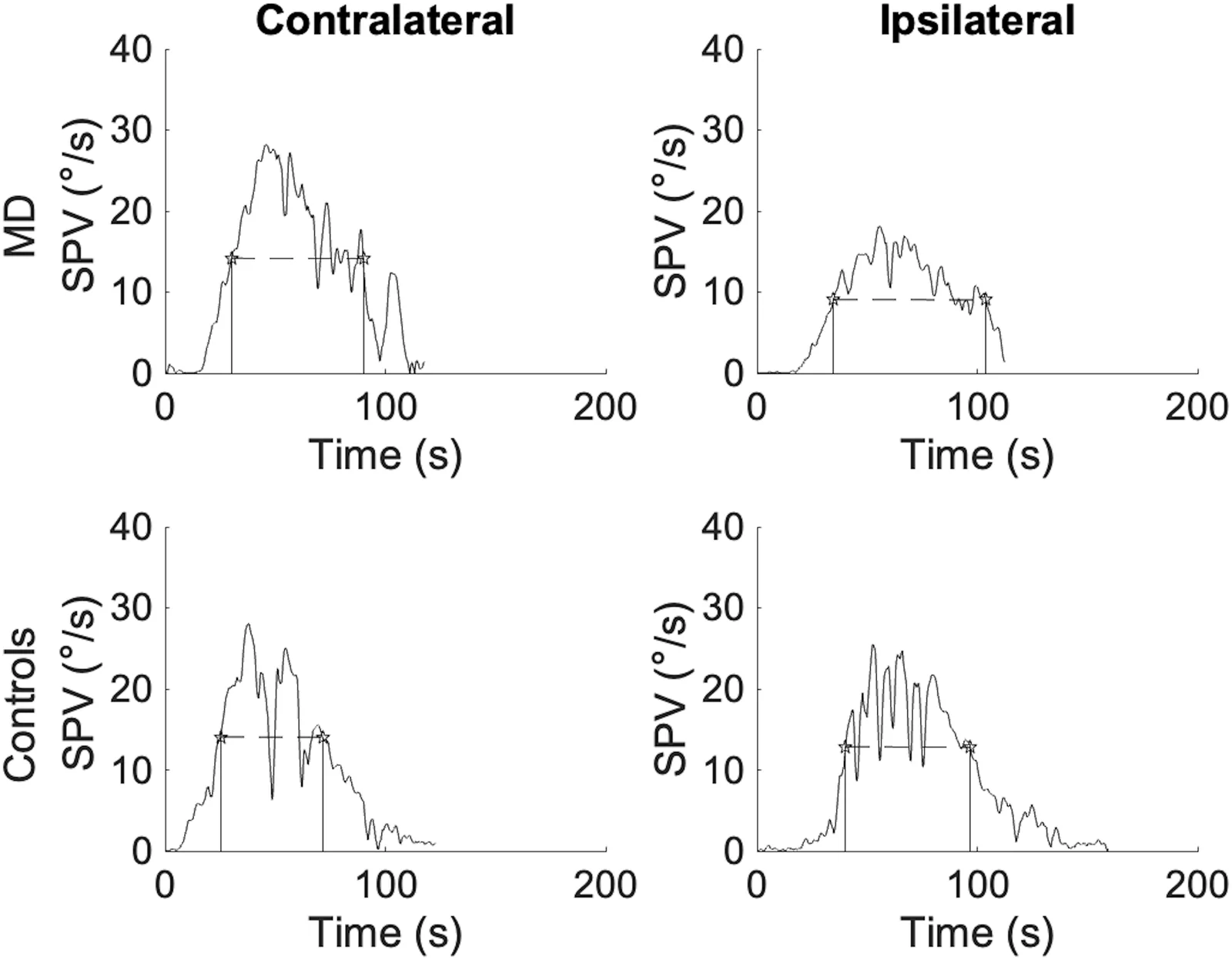
Raw data of a representative participant for each group (Meniere and Control) for both ears (ipsilateral and contralateral).

### Peak SPV

Comparing peak SPV following warm irrigation (Figure 3(a)), the results revealed a significant group difference (F (1, 22) = 6.32; p = 0.02; η_p_^2^ = 0.22). Additionally, a significant influence of ear condition was observed (F (1, 22) = 7.24; p < 0.01; η_p_^2^ = 0.25). However, no significant ear by groups interaction was identified (F (1, 22) = 2.80; p = 0.11; η_p_^2^ = 0.11). A post-hoc t-test using a Bonferroni correction revealed a significant difference between controls-contralateral and MD-ipsilateral ears (p = 0.02), and between controls-ipsilateral and MD-ipsilateral ears (p = 0.04). Additionally, significant differences were observed between MD-contralateral and MD-ipsilateral ears (p = 0.03).

**Figure 3.**
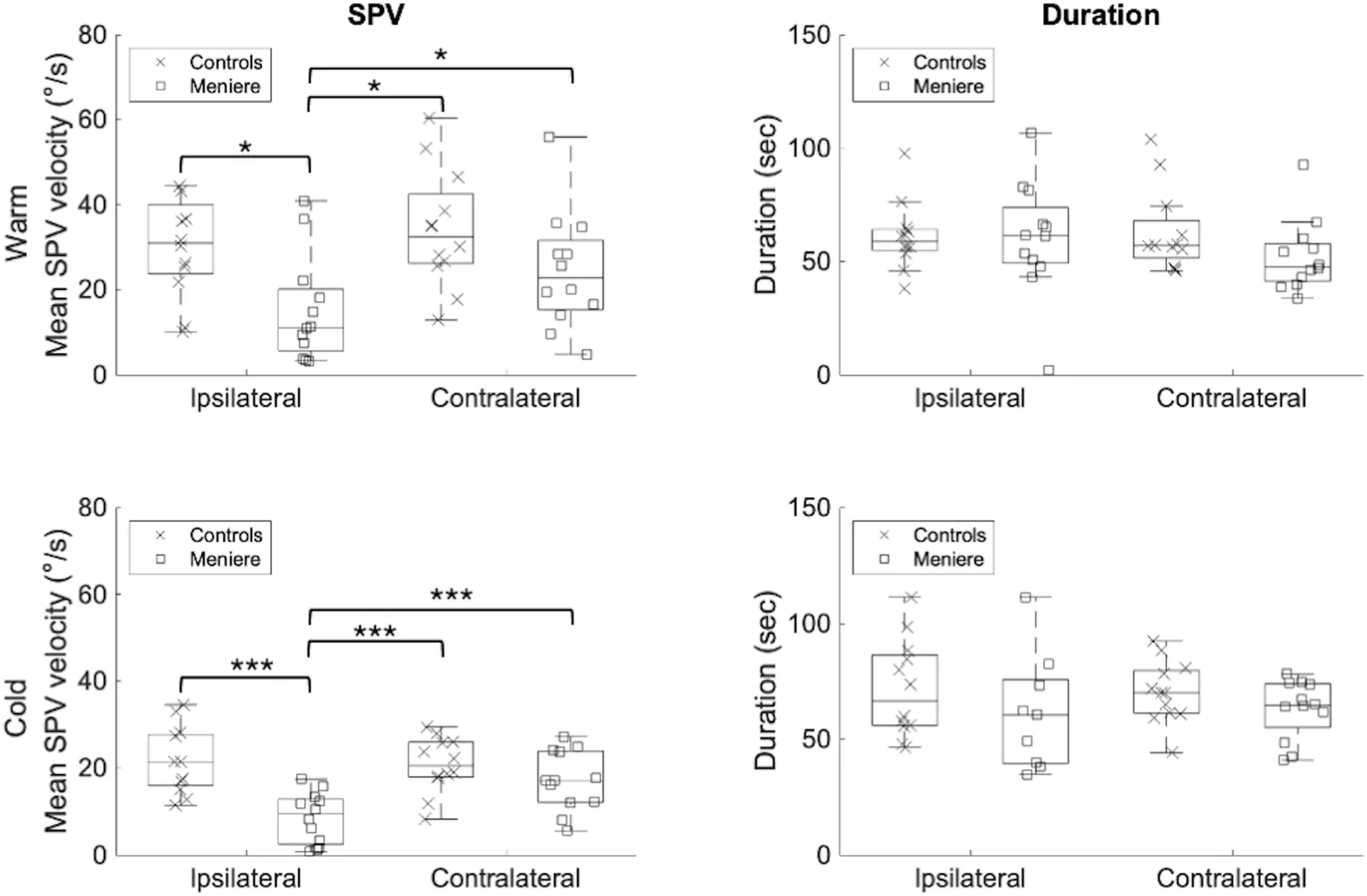
Boxplot representation of the SPV results (warm: A and cold: C) and nystagmus duration (warm: B and cold: D). Significant differences were observed between MD-ipsilateral ears and MD-contralateral as well as both groups of control ears following warm and cold irrigations for the SPV, but not for the duration. *p < 0.05; ***p < 0.001.

Comparing peak SPV following cold irrigation (Figure 3(c)), the results revealed a significant group difference (F (1, 22) = 10.56; p = 0.004; η_p_^2^ = 0.32). Additionally, a significant influence of ear condition (F (1, 22) = 16.45; p ≤ 0.001; η_p_^2^ = 0.428) and a significant ear by group interaction was observed (F (1, 22) = 26.96; p ≤ 0.001; η_p_^2^ = 0.55). A post-hoc t-test using a Bonferroni correction revealed significant difference between controls-contralateral and MD-ipsilateral ears (p ≤ 0.001), and between controls-ipsilateral and MD-ipsilateral ears (p ≤ 0.001). Additionally, significant differences were observed between MD-contralateral and MD-ipsilateral ears (p ≤ 0.001).

### Duration of nystagmus

Comparing duration of nystagmus following warm irrigation (Figure 3(b)) failed to demonstrate either a significant group effect (F (1, 22) = 1.02; p = 0.33; η^2^ = 0.04), or any significant influence of ear (F (1, 22) = 0.88; p = 0.36; η_p_^2^ = 0.04).

Similar findings were obtained when comparing the duration of nystagmus following cold irrigation (Figure 3(d)). Neither a significant group effect (F (1, 19) = 1.75; p = 0.2; η_p_^2^ = 0.08) nor a significant influence of ear was observed (F (1, 19) = 0.06; p = 0.81; η_p_^2^ = 0.003).

## Discussion

In the present study, the main objective was to compare the peak of the slow eye phase velocity (SPV) and the nystagmus duration in patients with and without Meniere’s disease. The study confirmed that SPV in MD-affected ears differs significantly from contralateral (MD-unaffected) ears and controls, independently of the irrigation temperature. These SPV results are consistent with previous studies demonstrating that patients with Meniere’s disease have unilateral caloric weakness (low SPV) on the side of the affected ear.^2,23^ Indeed, in Meniere’s disease, the presence of endolymphatic hydrops is thought to induce an enlargement of the membranous duct, which it is hypothesized to produce a local flow within the duct resulting in a reduction of the caloric reaction.^7,8^ However, the study also found that nystagmus duration does not significantly differ between MD-affected ears and both MD-unaffected or control ears. This suggests that nystagmus duration is not impacted by thermal property of the labyrinth as suggested by Henriksson,^16^ but more probably by direct effect of temperature on semicircular canal afferents.^24^ These results are consistent with other studies that found that caloric testing can produce nystagmus even if the semicircular canal is plugged and, when plugged, remains responsive independent of head position.^24,25^ Convection mechanisms having greater influence than direct temperature on the SPV could explain why nystagmus duration is the same in controls and MD patients even if MD patients have reduced SPV peak in the affected ear.

Another possibility that may explain the discrepancy between SPV and duration is that nystagmus duration may be determined primarily by central, rather than peripheral processes, and these central processes may not be altered in hydrops. Several drugs used for vestibular disorders such as Meniere’s disease have been evaluated for their effect on nystagmus produced by caloric testing.^26^ These studies showed that anticholinergic medications such as atropine, which have a significant effect on activity in the vestibular nuclei^27^ affect only the SPV. Amphetamine and caffeine, in contrast, have an impact only on the nystagmus duration, prolonging the time constant without changing the gain and do not seem to affect significantly the peripheral auditory and vestibular function.^28,29^ This evidence about drugs may support that nystagmus duration following caloric irrigation may be partially controlled by central structures.

One central mechanism that may play a role in the duration of nystagmus in Meniere’s patients, particularly during low-frequency vestibular stimulation such as caloric stimulation, is velocity storage.^13^ The velocity storage mechanism (VSM) is controlled by brainstem and cerebellar structures including the vestibular nuclei^30,31^ and consists of both “direct” and “indirect” pathways receiving input from the peripheral vestibular system. The “direct” pathway allows for rapid changes in eye position, while the “indirect” pathway has a slower response, with longer charge and discharge times compared to the “direct” pathway.^32^ The “indirect” pathway may contribute to the duration of nystagmus following caloric irrigation. In patients with Ménière’s disease (MD), the velocity storage mechanism (VSM) appears to remain functionally intact provided there is no hair cell damage, which may account for the absence of significant differences in nystagmus duration observed in these patients.^33^ However, caloric stimulation may not be the most appropriate method for evaluating the VSM.^34^ Alternative approaches, such as modifications to the protocol described by Formby and Robinson,^35^ may offer additional insight, as this modified caloric paradigm is designed to replicate a step response comparable to that elicited by rotary chair testing. Future studies should further examine the relationship between velocity storage and nystagmus duration following caloric stimulation.

This study has some limitation that need to be considered. First, some patients were excluded due to missing data. Second, nystagmus duration is more difficult to measure than SPV and previous work has struggled to define a consistent method. For example, some studies arbitrarily determined when the nystagmus started and when it stopped,^26^ while others predetermined a threshold to identify when nystagmus started and ended.^13,14^ In the present study, we used the duration at greater than half maximum amplitude method.^36^ We believe this method may be less subjective and prevent from interpreting artefacts as it accounts for the strength of the response. Future studies may further investigate methods to measure nystagmus duration to allow direct comparisons between studies. Future studies might also measure motion sensitivity and its relation to nystagmus duration following caloric stimulation.^37^

## Conclusion

The results support existing findings that SPV can be affected in patients with MD even if if the vHIT is normal. However, mechanisms that influence the nystagmus SPV seem to differ from those for the nystagmus duration. Nystagmus duration seems to remain unaffected in MD patients with vHIT-caloric dissociation, which suggest that this function may be controlled by other factors such as the velocity storage mechanism.^38^
